# Sensory processing of women diagnosed with genito-pelvic pain/penetration disorder: a research proposal

**DOI:** 10.1186/s13104-019-4612-6

**Published:** 2019-09-13

**Authors:** Elsie Labuschagne, Matty van Niekerk

**Affiliations:** Department of Occupational Therapy, School of Therapeutic Sciences, Faculty of Health Sciences, University of Witwatersrand, 7 York Road, Johannesburg, 2193 South Africa

**Keywords:** Sensory processing disorder, Genito-pelvic pain/penetration disorder, Female sexual pain, Anxiety, Occupational therapy, Sensory integration

## Abstract

**Objectives:**

The study objectives are to describe the sensory processing patterns of women diagnosed with genito-pelvic pain/penetration disorder (GPPPD), to explore the level of anxiety when both GPPPD and sensory processing disorder (SPD) are present and to investigate participants’ experience of participating in a sensory-based home programme.

**Methods:**

A descriptive two-phased study design will be used. Phase one is a quantitative, cross sectional non-experimental descriptive study, using the Adolescent/Adult Sensory History (ASH) and Hospital Anxiety and Depression Scales (HADS) to obtain data from purposive sampling. Phase two is an exploratory qualitative study involving participants who were identified with SPD in phase one. They will participate in a sensory-based home programme and their experience thereof will be established during semi-structured interviews.

**Outcomes:**

Descriptive studies are known to be useful in planning health services and to develop hypotheses for future testing. This study could improve practitioners’ understanding of GPPPD and SPD and make alternative, non-invasive, non-pharmacological treatment options available to better assist these patients. The study could further clarify the role of the occupational therapist in sexuality. Exploring participants’ anxiety has important implications for treatment protocols in occupational therapy and assisting in describing the signs and symptoms of GPPPD.

## Introduction

The role of sexuality is often ignored in occupational therapy. Literature regarding sensory processing and female sexual dysfunction, specifically genito-pelvic pain/penetration disorder (GPPPD), is virtually non-existent. In occupational therapy, sexuality falls into the performance of activities of daily living (ADL) as well as the fulfilment of various roles. Dysfunction in this performance area is therefore of concern to occupational therapists, as it may affect a client’s occupational performance, activity participation and thus well-being [1]. A study by Engel-Yeger et al. [2] recommends that occupational therapists address intimate relationships during sensory processing disorder (SPD)-related intervention with adults.

Women diagnosed with GPPPD experience sexual and psychological difficulties as well as significant relationship impairments [3]. The sensation of pain (which is linked to interoception [4, 5]) is an important symptom of GPPPD and is the only sensory modality that is commonly researched and explored. A recent study [6] found pain sensitivity to be related to over-responsivity in a person with sensory modulation disorder (SMD), a sub-component of SPD.

Sensory processing disorder (SPD) [7–9] is a result of difficulty grading and/or regulating responses to sensory input and collectively refers to three diagnostic groups, namely sensory modulation disorder (SMD), sensory discrimination disorder (SDD) and sensory-based motor disorder (SBMD) [7, 10]. The diagnostic groups and their respective sub-types [7, 10] are illustrated in Table 1.

**Table 1:** Sensory processing disorder: diagnostic groups and subtypes. Adapted from: (Miller et al. 2007: page 137) [8]

Diagnostic groups:	Sensory modulation disorder (SMD)	Sensory discrimination disorder (SDD)	Sensory based motor disorder (SBMD)
Subtypes	Sensory over-responsivity (SOR)	Auditory	Postural disorder
	Sensory under-responsivity (SUR)	Proprioception	Dyspraxia
	Sensory seeking/craving (SS)	Tactile	
		Taste/smell	
		Vestibular	
		Visual	

Individuals with SOR experience non-painful sensations as abnormally irritating, unpleasant or painful [7, 12], which can result in defensive behaviour, such as tactile defensiveness. Atypical sensory processing responses have significant implications for quality of life (QoL) [11, 13–15], pain [6, 16–19], socio-emotional aspects [20–24], interpersonal relationships [25–28], and intimacy [2, 29].

A multi-disciplinary, multi-modal approach [30–34] is emphasised for treatment of GPPPD. Current GPPPD treatment options range from medical intervention, physical therapy, and psychosocial treatments, which reflect the current concepts regarding its aetiology [33]. Physical touch is often emphasised in relationships [29], therefore many professionals introduce touch therapy (e.g. sensate focus) as a treatment modality for sexual dysfunction [34, 35]. However, conventional treatment methods used by the multi-disciplinary team to treat female sexual dysfunction may be rendered ineffective, or may actually exacerbate the condition in persons with SPD.

The possible inter-relatedness of GPPPD and SPD could have significant implications, not only in understanding SPD and its impact on intimate relationships, but also for conventional treatment methods used by the multi-disciplinary team. It could uncover another factor in the aetiology of female sexual pain and lay the foundation for inclusion of sensory integrative occupational therapy treatment in the current multi-modal approach.

Anxiety has been confirmed to accompany diagnoses of both sensory processing [20, 22, 23, 36] and sexual pain disorders [37–42]. The presence of SPD is not only a risk factor for the development of mental health conditions, e.g. anxiety, [6] but is also known to have an impact on the treatment of anxiety. The presence of sensory defensiveness hampers the treatment of mental health problems, e.g. anxiety, and pharmacological and psychological approaches only offer a short term solution [11]. A 2010 study of sensory defensiveness (a form of SOR) and mental health [11] found that treating anxiety through mainly cognitive strategies was ineffective in persons with SOR. Since tactile-based therapies have been shown to be effective at modulating arousal, attention and sensory defensiveness [43], these treatment modalities should be used in conjunction with traditional treatment of anxiety, particularly for people with SPD.

### Objectives

The objectives of the study are to describe the sensory processing patterns of women diagnosed with GPPPD, to explore the level of anxiety when both GPPPD and SPD are present and to investigate participants’ experience of participating in a sensory-based home programme.

## Main text

### Study design

Since there is a dearth of information related to SPD and GPPPD in combination, a descriptive two-phased study [44] will be conducted.

Phase one consists of a quantitative, non-experimental descriptive study. Online questionnaires will be used to collect data regarding participants’ sensory processing.

Phase two consists of a qualitative study using semi-structured individual interviews to gather information regarding participants’ experience of participating in a sensory- based home program. Qualitative research designs can, however, evolve and may only be finalised once data collection ends [44].

### Phase one

#### Participants

Sex, intimacy and sexual pain remain private topics which may result in a reluctance to participate. In an attempt to overcome this barrier, healthcare professionals (HCP) working in the field of sexual health will be recruited to invite their patients to participate in the study, which introduces omission bias, as potential participants in the public sector are likely to be excluded. Purposive sampling [45, 46] with snowballing will be used in an attempt to overcome the omission bias. All women who meet the inclusion criteria will be included. The estimated number of patients seen per annum by healthcare professionals consulted prior to commencing the study indicated a population size of 200 women. In order for the survey results to be statistically valid a minimum of 132 completed questionnaires are required. The margin of error was set at 5% and the confidence level at 95%. With an estimated response rate of 25% for online surveys, a total of 528 participants would have to be invited to achieve the required sample size. Inclusion criteria are (i) females from the age of 18 and (ii) a diagnosis of GPPPD. Exclusion criteria are (i) previous treatment for SPD; (ii) diagnosis affecting the neurological system e.g., Multiple Sclerosis; (iii) cancer-related diagnosis; and (iv) pregnant at time of completing questionnaire.

#### Research instruments

##### Sensory processing

Sensory processing patterns will be measured by 163 items on the Adolescent/Adult Sensory History (ASH) questionnaire [47]. Reliability of the ASH’s total score is 0.85 and concurrent validity 0.78 (p < 0.001) [47–49]. The ASH is relatively new, but has already been used in a few studies [50, 51]. This self-report questionnaire identifies dysfunction in five key areas, namely sensory discrimination, sensory modulation, postural ocular skills, praxis/motor coordination, and social-emotional functioning, [47] as well as functional problems related to each of these areas. Additionally, it describes overall sensory processing which is also divided into sub-sections based on sensory modalities (e.g., touch and taste). It uses a five-point Likert-type scale and can be used by individuals aged 13 to 95 years. The questionnaire takes 15 to 20 min to complete.

The ASH provides a total score, reflecting the functioning in overall sensory processing. This is followed by sub-scores for the sensory section, sensory modulation, sensory discrimination, functional problems and motor/social sections respectively. Each sub-score also consists of separate modalities which identify problems in specific areas.

While the original instrument allows for a small number of questions not to be completed, the online questionnaire will only proceed to the next section if all items have been completed, thus attempting to minimise incomplete questionnaires which cannot be included for analysis.

##### Anxiety

Levels of anxiety will be assessed by the Hospital Anxiety and Depression Scale’s (HADS) scales for Anxiety (HADS-A). This self-administered subscale consists of seven questions for anxiety, with a four-point (0 to 3) ordinal response format. The instrument takes between two to five minutes to complete. The HADS-A has a correlation score of 0.80 and the validity has been described as good to very good [52]. Cut-off scores are available for quantification, for example a score of 8 or more for anxiety has a specificity of 0.78 and a sensitivity of 0.9, and for depression a specificity of 0.79 and a sensitivity of 0.83 [53, 54].

Cut-off scores existed for the following diagnostic categories: normal (score 0 to 7), borderline (8 to 10) and clinical/abnormal (score 11 to 21).

#### Procedure/data collection

Potential participants will receive an e-mail from the HCP containing information regarding SPD and a link to a secure online questionnaire on the Research electronic data capture (REDCap) platform. Participation in both phases will be completely voluntary and participants can withdraw from the study without any detriment at any time. Informed consent will be obtained electronically, prior to completing the demographic information (age, gender, additional diagnosis, highest qualification, marital status, number of children and age, sexual history, province) and online questionnaires (ASH and HADS). Data can be submitted anonymously, or an e-mail address can be provided should participants wish to be considered for phase two of the study. Each online questionnaire will receive a unique identification number. All identifying data (including consent regarding participation in phase two and e-mail addresses) will be kept securely, separate from the questionnaires. Once a questionnaire has been scored and SPD identified, the questionnaire number will be compared to the list of participants who gave consent to be contacted regarding participation in phase two.

Ethical clearance has been obtained from the Human Ethics Research Committee at the University of the Witwatersrand (Certificate Number M170829).

#### Data analysis

Responses to the questions will be assigned a numeric value within REDCap software. The raw data for each participant will be exported from REDCap to Excel.

The responses to the ASH will be entered into the AASH-Scoring Program©, which generates individual reports providing raw scores, z-scores and interpretation of scores. Standardised scores will be divided into three categories (nominal variables), namely “typical performance”, “mild difficulties” (frequently demonstrates functional difficulties in some areas of sensory, motor or social/emotional processing) and “definite difficulties” (performance is well outside typical performance and almost always results in functional difficulties). Clinically, a diagnosis of “mild difficulty” requires further investigation or assessment whereas a diagnosis of “definite difficulty” requires intervention. For analysis purposes, the “mild difficulty” and “definite difficulty” categories will be combined, to indicate the percentage of participants who fall outside the parameters of typical functioning.

The responses to the HADS-A will be exported to Excel and scored according to its categories, namely normal, borderline and clinical/abnormal.

Results obtained on the ASH and HADS-A (only anxiety items will be computed as the presence of depression is beyond the scope of the current study) will be transferred to Excel for each participant and then to an Excel summary sheet for each instrument. The cleaned data will be imported into Statistica analytics software program.

Descriptive statistics will be used to analyse data. Ordinal data obtained via the demographic questionnaire, specifically age and sexual history will be analysed using measurement of central tendency, specifically the mean and median, as well as measurement of dispersion of the data, specifically the standard deviation and range.

Categorical data obtained via the ASH and HADS-A will be analysed using frequencies and percentages.

### Phase two

#### Participants

Purposive sampling will be used and participants who meet the inclusion criteria of phase two will be invited to participate in a sensory-based home program. Inclusion criteria are (i) a diagnosis of SPD identified in phase one; (ii) consent to take part in phase two; and (iii) resides in Gauteng or Kwazulu-Natal provinces to attend interviews.

#### Procedure/data collection

The sensory-based home-programme consists of an initial interview, followed by implementation of strategies and a follow-up interview.

Qualitative data will be collected through initial individual interviews, during which information regarding personal experiences and impact of sensory processing, SPD, personalised interventions, and possible treatment techniques will be discussed and/or demonstrated. The home programme will be client-centred and may include interventions, such as changes to the environment, adaptations to tasks, preventing and/or avoiding potential sensory triggers, as well as self-regulation strategies. The researcher will be available telephonically should any questions arise during the execution of the home programme. Semi-structured, individual follow-up interviews will be conducted to obtain information regarding participants’ experience of participating in a sensory-based home programme. The follow-up interview may be conducted via electronic media e.g., Skype. All interviews will be audio-recorded and transcribed. The researcher will take field notes during the interviews. Data will be collected until saturation is reached and no new information is obtained during the interviews. According to Guest et al. [45] six to twelve interviews should suffice when the aim is to describe a homogeneous group’s perceptions and experiences using nonprobability sampling. Malterud et al. [55] introduced the model of “information power” for qualitative studies where sample size is determined by the amount of relevant information related to the research question. Data collected will be checked on an ongoing basis and these sampling methods will assist in determining the sample size/data saturation [56].

#### Data analysis

Inductive thematic content analysis [57–59] will be used to analyse data obtained from the interviews. A computer-assisted qualitative data analysis software program, Atlas.ti8, will be used to analyse qualitative data. Vertical analysis of the individual transcripts will be done, and data will be grouped according to themes/topics identified, resulting in codes that capture the essential elements in the data. An inductive thematic network approach will be used, utilising a coding framework containing a list of codes emerging from the data. Codes could be added to the list or changed as the process unfolds. Once all the data have been coded, codes will be grouped into themes. Horizontal analysis will be used to look for common threads [56, 60].

Various parameters of trustworthiness will be applied to ensure rigor. This includes using an interview protocol [60] consisting of a list of open-ended questions supporting the research question, to ensure a consistent style of data collection. The implementation of the sensory-based home programme will follow a standard framework, but include individual treatment activities based on participants’ unique sensory processing profile obtained from phase one. Furthermore, the researcher will ensure prolonged engagement in the field, thus data will be collected until saturation is reached and no new information is obtained during the interviews. An inductive thematic saturation model will be used to ensure saturation [61]. Reflexivity will be practised throughout the process and continuous self-examination will be done to ensure that researcher-subjectivity does not interfere with data collection. This will also assist in limiting bias during data collection and contribute to the quality and objectivity of the results.

Findings will be reported using thick, rich descriptions of data, ensuring validity [56, 60]. Member checking will be done to ensure data were interpreted accurately [62].

An audit trail will assist with checking procedures followed and conclusions reached, enhancing credibility and dependability of the results. Dependability of data will be further enhanced by peer coding. Codes will be checked with an independent person, i.e. the researcher’s supervisor.

A data management system is crucial to rigorous qualitative research. All records and data will be managed, maintained and backed-up using the Office365 cloud, with assistance from the University’s data librarian. Once the study has been completed, the data will be stored in the University library’s research repository.

## Conclusion

This is to our knowledge the first study investigating the sensory processing patterns of women diagnosed with GPPPD.

Although much has been written about GPPPD and SPD as separate entities, there is a paucity of literature describing the potential co-existence of these two conditions. The study results could be used to assist in planning healthcare services for women with GPPPD and to develop hypotheses for future research [63]. Since a multidisciplinary and multidimensional approach is already recommended for treating GPPPD, describing the sensory processing patterns of women with GPPPD may have important implications for future treatment of GPPPD. Sensory processing intervention has not been evaluated as a possible option for managing GPPPD, despite evidence suggesting that SPD intervention assists with pain management [18]. Current conventional, multi-disciplinary treatment methods for female sexual dysfunction, e.g. sensate focus, may be rendered ineffective or actually worsen the condition in persons with SPD, thus necessitating identifying the sensory processing patterns of women with GPPPD prior to commencing treatment.

A common theme in both these study fields is the presence of affective symptoms, i.e. anxiety in women diagnosed with a sexual disorder [38], or adults diagnosed with SPD [64], necessitating investigating the extent of participants’ anxiety.

## Limitations

SPD is not necessarily a well-known field outside of occupational therapy, thus a lack of knowledge regarding SPD among participants and referring HCPs could result in non-participation because HCPs and/or women with GPPPD do not grasp the value of understanding sensory processing patterns and its importance in managing pain (and thus potentially GPPPD). Non-participation may give rise to sampling bias. In an attempt to overcome this aspect of selection bias, the researchers developed a concise information sheet describing SPD, to educate both the HCPs and the participants.

Since phase one data are obtained via self-report questionnaires, participants may want to give socially desired responses which could give rise to measurement bias. This was ameliorated by ensuring anonymity. Providing socially desired responses on the ASH could result in misinterpreting sensory processing difficulties. Thus, using the ASH and interpreting it carefully will assist in mitigating measurement bias in cases where participants agreed to be identified for inclusion in phase two.

Study sample size may be limited due to purposive sampling, because recruitment will be done by HCPs practicing in the field of sexual health. Thus the sample is potentially limited to clients with access to private medical facilities and more extensive personal resources. For this reason, snow-ball sampling was included to encourage both HPCs and participants to include practitioners/participants from the public health sector.

Voluntary participation may also impact on sample size as sensitivity and possible stigma surrounding sexual dysfunction may limit study participants to those whose symptoms are severe enough to seek help. Very anxious/sensitive clients may not be accessed due to their fear of seeking help. Phase two’s sample size may be further affected by clients’ willingness to take part in a sensory-based home programme and limited participants may be available in the provinces indicated.

However, despite these limitations, this is an important study due to the dearth of information and thus will potentially make a valuable contribution to the body of knowledge.

## Data Availability

Not applicable. This is a research proposal and no datasets were generated.

## Abbreviations

ASH: Adolescent/Adult Sensory History
ADL: activities of daily living
GPPPD: genito-pelvic pain/penetration disorder
HADS: Hospital Anxiety and Depression Questionnaire
HADS-A: Hospital Anxiety and Depression Scale’s scales for Anxiety
HCP: Healthcare professionals
QoL: quality of life
REDCap: research electronic data capture
SBMD: sensory based motor disorder
SDD: sensory discrimination disorder
SMD: sensory modulation disorder
SOR: sensory over-responsivity
SPD: sensory processing disorder
SS: sensory seeking
SUR: sensory under-responsivity
